# Supplementary material for: E. coli TraR allosterically regulates transcription initiation by altering RNA polymerase conformation
Source: eLife. 2019 Dec 16;8:e49375. doi: 10.7554/eLife.49375 (PMC6970531; doi:10.7554/eLife.49375)
Supplement: Supplementary file 1. [file elife-49375-supp1.docx]

**Supplementary file 1.** Cryo-EM data acquisition and refinement parameters.

| **Sample** | Eσ^70^ |  | TraR-Eσ^70^(I) | TraR-Eσ^70^(II) | TraR-Eσ^70^(III) |  | *rpsT* P2-RPo | *rpsT* P2-RPo2 |
| --- | --- | --- | --- | --- | --- | --- | --- | --- |
| **EMDB** | EMD-20230 |  | EMD-0348 | EMD-0349 | EMD-20231 |  | EMD-20203 | EMD-20232 |
| **PDB** | 6P1K |  | 6N57 | 6N58 | - |  | 6OUL | - |
|  |  |  |  |  |  |  |  |  |
| **Data collection and processing** |  |  |  |  |  |  |  |  |
| Microscope | Talos Arctica |  | FEI Titan Krios | | |  | FEI Titan Krios | |
| Voltage (kV) | 200 |  | 300 | | |  | 300 | |
| Detector | K2 summit |  | K2 summit | | |  | K2 summit | |
| Electron exposure (e–/Å2) | 37.3 |  | 71 | | |  | 47.3 | |
| Defocus range (μm) | 1.0-2.5 |  | 0.8-2.4 | | |  | 0.5-2.5 | |
| Data collection mode | Counting |  | Counting | | |  | Super-resolution | |
| Nominal Magnification | 28,000x |  | 22,500x | | |  | 22,500x | |
| Pixel size (Å) | 1.5 |  | 1.3 | | |  | 1.3 | |
| Symmetry imposed | C1 |  | C1 | | |  | C1 | |
| Initial particle images (no.) | 1387166 |  | 101,0867 | | |  | 973,481 | |
| Final particle images (no.) | 358,725 |  | 153,295 | 123,607 | 95,767 |  | 289,670 | 46,378 |
| Map resolution (Å) - FSC threshold 0.143 | 4.05 |  | 3.7 | 3.78 | 3.91 |  | 3.43 | 3.91 |
| Map resolution range (Å) | 3.4-8.5 |  | 3.1-7.0 | 3.2-7.0 | 3.5-8.5 |  | 3.3-8.0 | 2.9-8.8 |
|  |  |  |  |  |  |  |  |  |
| **Refinement^c^** |  |  |  |  |  |  |  |  |
| Initial model used (PDB code) | 4LJZ (EΔ1.1σ^70^)^a^  4LK1 (σ^70^_1.1_)^a^ |  | 4LJZ (EΔ1.1σ^70^)^a^  4LK1 (σ^70^_1.1_)^a^  5W1S (TraR)^b^ | |  |  | 4LJZ (EΔ1.1σ^70^)^a^ |  |
| Map sharpening B factor (Å2) | -194 |  | -95 | -94 | -79 |  | -95 | -66 |
| Model composition |  |  |  |  |  |  |  |  |
| Non-hydrogen atoms | 29,160 |  | 30,124 | 30,188 |  |  | 31,926 |  |
| Protein residues | 3,714 |  | 3,841 | 3,833 |  |  | 3,751 |  |
| Nucleic acid residues | 0 |  | 0 | 0 |  |  | 117 |  |
| Ligands | 1 Mg^2+^  2 Zn^2+^ |  | 1 Mg^2+^  3 Zn^2+^  4 CHAPSO | 1 Mg^2+^  3 Zn^2+^  4 CHAPSO |  |  | 1 Mg^2+^  2 Zn^2+^  3 CHAPSO |  |
| B factors (Å2) |  |  |  |  |  |  |  |  |
| Protein | 37.68 |  | 47.80 | 39.95 |  |  | 36.90 |  |
| Nucleic acid | - |  | - | - |  |  | 106.08 |  |
| Ligands | 49.76 |  | 51.05 | 42.64 |  |  | 34.18 |  |
| R.m.s. deviations |  |  |  |  |  |  |  |  |
| Bond lengths (Å) | 0.008 |  | 0.011 | 0.012 |  |  | 0.014 |  |
| Bond angles (°) | 1.131 |  | 1.062 | 1.147 |  |  | 1.130 |  |
| Validation |  |  |  |  |  |  |  |  |
| MolProbity score | 1.75 |  | 2.19 | 2.14 |  |  | 2.07 |  |
| Clashscore | 3.60 |  | 9.67 | 8.96 |  |  | 8.43 |  |
| Poor rotamers (%) | 0.66 |  | 0.25 | 0.37 |  |  | 0.31 |  |
| Ramachandran plot |  |  |  |  |  |  |  |  |
| Favored (%) | 88.16 |  | 83.86 | 84.92 |  |  | 87.50 |  |
| Disallowed (%) | 0.16 |  | 0.29 | 0.37 |  |  | 0.27 |  |

^a^(Bae et al., 2013)

^b^(Molodtsov et al., 2018)

^c^Refinement: PHENIX real_space_refine (Adams et al., 2010). Validation: MOLPROBITY (Chen et al., 2010).
