# Supplementary material for: E. coli TraR allosterically regulates transcription initiation by altering RNA polymerase conformation
Source: eLife. 2019 Dec 16;8:e49375. doi: 10.7554/eLife.49375 (PMC6970531; doi:10.7554/eLife.49375)
Supplement: Supplementary file 2. [file elife-49375-supp2.docx]

**Supplementary file 2.** Details of flux calculator (Galburt, 2018) calculations.

| constants | | inhibited promoter | activated promoter |  | inhibited promoter | activated promoter |
| --- | --- | --- | --- | --- | --- | --- |
| Galburt (2018) nomenclature | current nomenclature | basal = WT-RNAP reg = Δ1.1σ^70^-RNAP  (Figure 4 - figure supplement 1C) | |  | basal = no TraR  reg = TraR  (Figure 8) | |
| k^basal^_on_ (nM^-1^s^-1^) | k^basal^_2_ (s^-1^) | 1 | 0.1 |  | 1 | 0.1 |
| k^reg^_on_ (s^-1^) | k^reg^_2_ (s^-1^) | 1 | 0.1 |  | 5 | 0.5 |
| k^basal^_/_k^reg^ | | 1 | 1 |  | 5 | 5 |
|  |  |  |  |  |  |  |
| k^basal^_off_ (s^-1^) | k^basal^_-2_ (s^-1^) | 10 | 1 |  | 10 | 1 |
| k^reg^_off_ (s^-1^) | k^reg^_-2_ (s^-1^) | 10 | 1 |  | 10 | 1 |
| k^basal^_/_k^reg^ | | 1 | 1 |  | 1 | 1 |
|  |  |  |  |  |  |  |
| k^basal^_open_ (s^-1^) | k^basal^_3_ (s^-1^) | 1 | 0.1 |  | 1 | 0.1 |
| k^reg^_open_ (s^-1^) | k^reg^_3_ (s^-1^) | 25 | 1 |  | 10 | 1 |
| k^basal^_/_k^reg^ | | 25 | 10 |  | 10 | 10 |
|  |  |  |  |  |  |  |
| k^basal^_close_ (s^-1^) | k^basal^_-3_ (s^-1^) | 50 | 0.05 |  | 50 | 0.05 |
| k^reg^_close_ (s^-1^) | k^reg^_-3_ (s^-1^) | 1300 | 1.3 |  | 2500 | 2.5 |
| k^basal^_/_k^reg^ | | 26 | 26 |  | 50 | 50 |
|  |  |  |  |  |  |  |
| k^basal^_esc_ (s^-1^) | k^basal^_4_ (s^-1^) | 5 | 5 |  | 5 | 5 |
| k^reg^_esc_ (s^-1^) | k^reg^_4_ (s^-1^) | 5 | 5 |  | 5 | 5 |
| k^basal^_/_k^reg^ | | 1 | 1 |  | 1 | 1 |
|  | |  |  |  |  |  |
| flux^basal^ (min^-1^) | | 5.3 | 5.8 |  | 5.3 | 5.8 |
| flux^reg^ (min^-1^) | | 5.6 | 83 |  | 1.2 | 35 |
| flux ratio (basal/reg) | | 1.06 | 14.3 |  | 0.23 | 6.0 |
