# Supplementary material for: E. coli TraR allosterically regulates transcription initiation by altering RNA polymerase conformation
Source: eLife. 2019 Dec 16;8:e49375. doi: 10.7554/eLife.49375 (PMC6970531; doi:10.7554/eLife.49375)
Supplement: Supplementary file 3. [file elife-49375-supp3.docx]

**Supplementary file 3**. RNAP conformational changes.

| structure | reference  structure | PyMOL align^a^ | PyMOL rms_cur^b^ | | | | |  | align | | rms_cur |
| --- | --- | --- | --- | --- | --- | --- | --- | --- | --- | --- | --- |
|  |  | structural  core^c^ | structural core | overall  (-structural core) | clamp^d^ | β'shelf-jaw^e^ | βlobe-Si1^f^ |  | BH^g^ | | |
| Eσ^70^ | RPo | 0.647 Å  (1273 Cαs) | 0.839 Å  (1356 Cαs) | 2.480  (1804) | 3.396  3.3°  open | 1.735  2.7°  twisted open | 2.912  3.4°  DNA-> |  | 0.484  (33) | 0.55  (35) | |
| TraR-Eσ^70^(I) | RPo | 0.454  (1249) | 0.673  (1380) | 4.931  (1817) | 2.880  3.6°  open | 2.675  4.1°  open | 10.213  18°  ->TraR |  | 0.81  (30) | 1.478  (35) | |
|  | H |  | 0.873  (1355) | 4.567  (1806) | 1.413  1.8°  roll-CCW | 1.755  2.7°  open | 9.993  17°  ->TraR |  | 0.764  (30) | 1.485  (35) | |
| PDB 5w1s-A (TraR-Eσ^70^ complex A)^h^ | TraR1 | 0.632  (1251) | 0.871  (1377) | 6.323  (1646) | 3.837  5.7°  open | 2.523  3.2°  closed | 13.968  24°  TraR-> |  |  |  | |
|  | RPo |  | 0.604  (1256) | 3.354  (1638) | 5.906  7.8°  open | 2.211  1.8°  twisted open | 3.912  6.1°  TraR-> |  | 0.585  (35) | 0.585  (35) | |
|  | H |  | 0.680  (1241) | 2.699  (1629) | 3.226  4.4°  open | 2.030  0.86° | 5.007  8.1°  TraR-> |  |  |  | |
|  | 4yg2a | 0.344  (1306) | 0.541  (1482) | 1.089  (1648) | 1.587  1.7°  open | 1.174  1.2° | 1.351  2.4°  TraR-> |  |  |  | |
| PDB 5w1s-B (TraR-Eσ^70^ complex B)^h^ | TraR1 | 0.731  (1279) | 0.963  (1369) | 5.780  (1637) | 2.583  4.8°  open | 2.948  3.7°  closed | 12.498  22°  TraR-> |  |  |  | |
|  | RPo |  | 0.938  (1370) | 2.865  (1635) | 4.376  5.7°  open | 2.826  3.9°  twisted | 2.955  5.7°  DNA-> |  | 0.509  (33) | 0.576  (35) | |
|  | H |  | 0.989  (1355) | 2.7  (1627) | 2.256  3.4°  roll-CCW | 2.365  1.8°  closed | 4.998  8.8°  TraR-> |  |  |  | |
|  | 4yg2b | 0.358  (1283) | 0.47  (1372) | 0.73  (1638) | 0.864  0.89° | 0.887  1.2° | 0.622  1.2° |  |  |  | |
| PDB 4yg2-A (Eσ^70^ complex A)^i^ | RPo |  |  |  |  |  |  |  | 0.519  (35) | 0.519  (35) | |
| PDB 4yg2-B (Eσ^70^ complex B)^i^ | RPo |  |  |  |  |  |  |  | 0.415  (31) | 0.568  (35) | |

^a^The structure in the first column (structure) was aligned to the structure in the second column (reference structure) by Cα atoms only using the PyMOL align command, which superimposes the two structures using an algorithm that rejects outliers (such as in flexible loops, etc.) to minimize the root-mean-square deviation (rmsd) while maximizing the number of aligned atoms. The entries list the resulting rmsd (Å, top row) and the number of Cα atoms aligned (in parentheses). For the clamp, βlobe-Si1, and β'shelf-jaw entries, the rotational angle and axis between the elements was calculated using the PyMOL script draw_rotation_axis.py (<https://pymolwiki.org/index.php/RotationAxis>). The rotation angle is listed in the second row. The direction of movement (from the target structure to the reference structure) is denoted below.

^b^The structures/structural elements denoted were compared (but not aligned) by Cα atoms only using the PyMOL rms_cur command, which calculates the rmsd for all of the specified atoms (no outliers removed).

^c^*Eco* RNAP structural core: αI, αII, β (1-27, 147-152, 445-455, 520-713, 786-828, 1060-1240), β' (343-368, 421-786), ω.

^d^*Eco* RNAP clamp: β (1319-1342), β' (1-342, 1318-1344), σ^70^ (92-137, 353-449).

^e^*Eco* RNAP β'shelf-jaw: β' (787-931, 1135-1315).

^f^*Eco* RNAP βlobe-Si1: β (153-444).

^g^*Eco* RNAP BH: β' (769-803).

^h^(Molodtsov et al., 2018).

^i^(Murakami, 2013).
