# Supplementary material for: E. coli TraR allosterically regulates transcription initiation by altering RNA polymerase conformation
Source: eLife. 2019 Dec 16;8:e49375. doi: 10.7554/eLife.49375 (PMC6970531; doi:10.7554/eLife.49375)
Supplement: Supplementary file 4. [file elife-49375-supp4.docx]

**Supplementary file 4.** Plasmids.

| **Plasmid** | **Genotype** | **Source** |
| --- | --- | --- |
| pRLG770 | In vitro transcription vector, AmpR | *Ross et al., 1990* |
| pRLG13065 | pRLG770 with *rrnB* P1 (−88 to +50) | *Ross et al.,2016* |
| pRLG13098 | pRLG770 with *argI* (−45 to +32) | *Barker et al.,2001* |
| pRLG13099 | pRLG770 with *hisG* (−60 to +1) | *Paul et al.,2005* |
| pRLG14658 | pRLG770 with *rpsT* P2 (−89 to +50) | *Lemke et al., 2011* |
| pRLG15142 | pET28a-His_10_-SUMO *traR*, KanR | Present study |
| pRLG15276 | pRLG770 with *thrABC* (-72 to +16) | *Barker et al.,2001* |
| pIA331 | pT7 αββ'(Δ943-1130) (∆Si3 RNAP) | *Artsimovitch et al., 2003* |
| pRLG15299 | *rpoB* Δ225–343ΩGG (∆Si1 RNAP) | *Gopalkrishnan et al., 2017* |
| pRLG14844 | pET28a-His_6_ P43A *traR*, KanR | *Gopalkrishnan et al., 2017* |
| pRLG14846 | pET28a- His_6_ P45A *traR*, KanR | *Gopalkrishnan et al., 2017* |
| pRLG14847 | pET28a-His_6_ E46A *traR*, KanR | *Gopalkrishnan et al., 2017* |
| pRLG15278 | pET28a-His_10_-SUMO R49A *traR*, KanR | Present study |
| pRLG15279 | pET28a-His_10_-SUMO K50A *traR*, KanR | Present study |
| pEcrpoABC(-XH)Z | Encodes *Eco* RNAP RpoA, B, C-PPX-His_10_, Z, AmpR | *Twist et al., 2011* |
| pACYCDuet-1_Ec_rpoZ | Encodes *Eco* RNAP rpoZ, CamR | *Twist et al., 2011* |
| pSAD1403 | pET28a-His_10_-SUMO *rpoD*, KanR | *Chen et al., 2017* |
| pSAD1406 | pET28a-His_10_-SUMO *rpoD (*∆1-93*) (*∆1.1 σ^70^), KanR | *Chen et al., 2017* |
