# Supplementary material for: E. coli TraR allosterically regulates transcription initiation by altering RNA polymerase conformation
Source: eLife. 2019 Dec 16;8:e49375. doi: 10.7554/eLife.49375 (PMC6970531; doi:10.7554/eLife.49375)
Supplement: Supplementary file 5. [file elife-49375-supp5.docx]

**Supplementary file 5.** Oligonucleotides and Geneblock sequences.

| **Primer** | **Sequence** |
| --- | --- |
| P43A *traR* | 5’ GAAGCATGCGGAAATGCTATTCCGGAAGCC |
| P45A *traR* | GGAAATCCTATTGCGGAAGCCCGGCGG |
| E46A *traR* | GGAAATCCTATTCCGGCAGCCCGGCGGAAAATA |
| R49A *traR* | ATTCCGGAAGCCCGGGCGAAAATATTTCCCGGT |
| K50A *traR* | ATTCCGGAAGCCCGGCGGGCAATATTTCCCGGT |
| SumoF | GGGGAATTGTGAGCGGATAACAATTCC |
| SumoR | GTCCCATTCGCCAATCCGGATATAG |
| TraR_sumo_vector_FOR | 5’- AAACATTATGCATAACAAAGCCCGAAAGGAAGC TGAG -3’ |
| pETsumo_traR_vector_REV | 5’- CGGCTTCATCACTTCCACCAATCTGTTCTCTGT GAGCC -3’ |
| TraR_sumo_fragment_REV | 5’- TCGGGCTTTGTTATGCATAATGTTTTCTCTGTC TTTCCTGATACG -3’ |
| TraR_sumo_fragment_FOR | 5’- CAGATTGGTGGAAGTGATGAAGCCGATGAAGCA TATTCAG -3’ |
| rrnBP1(-63_to+20)_top | 5’- GGTCAGAAAATTATTTTAAATTTCCTCTTGTCA GGCCGGAATAACTCCCTATAATGCGCCACCACTGAC ACGGAACAACGGCG -3’ |
| rrnBP1(-63_to+20)_bot | 5’- CGCCGTTGTTCCGTGTCAGTGGTGGCGCATTAT AGGGAGTTATTCCGGCCTGACAAGAGGAAATTTAAA ATAATTTTCTGACC -3’ |
| rpsTP2(-60to+25)_top | 5’- GGCGGCGCTTATTTGCACAAATCCATTGACAAA AGAAGGCTAAAAGGGCATATTCCTCGGCCTTTGAAT TGTCCATATAGAACGC -3’ |
| rpsTP2 (-60to+25)_bot | 5’- GCGTTCTATATGGACAATTCAAAGGCCGAGGAA TAT GCCCTTTTAGCCTTCTTTTGTCAATGGATTTGT GCAAATAAGCGCCGCC -3’ |
